# Association of Air Pollution and Socioeconomic Deprivation with Depression and Pain

**DOI:** 10.3390/ijerph23050543

**Published:** 2026-04-22

**Authors:** Jamie-Nicole Luistro, Melanie C. Kos, Cooper J. Sharp, Maya R. Kilcullen, Abraham Dachs, Isabell James, Ashley Dian Hawk, Jeremy Mennis, Ingrid R. Olson, David V. Smith

**Affiliations:** 1 Department of Psychology and Neuroscience, Temple University, Philadelphia, PA 19122, USA; melanie.kos@temple.edu (M.C.K.); cooper.sharp@temple.edu (C.J.S.); maya.kilcullen@temple.edu (M.R.K.); abraham.dachs@temple.edu (A.D.); is.jams45@gmail.com (I.J.); ashley.hawk@temple.edu (A.D.H.); ingrid.olson@temple.edu (I.R.O.); 2 Department of Geography, Environment and Urban Studies, Temple University, Philadelphia, PA 19122, USA; jmennis@temple.edu

**Keywords:** PM_2.5_, neighborhood deprivation, relative deprivation, depression, pain, aging

## Abstract

**Highlights:**

**Public health relevance—How does this work relate to a public health issue?**

- This work focuses on the associations of environmental stressors and socioeconomic deprivation with individuals’ mental and physical health.

**Public health significance—Why is this work of significance to public health?**

- Our findings clarify the role of multiple levels of socioeconomic deprivation on experiences of depression and pain.
- Age significantly interacted with our measures of socioeconomic deprivation, emphasizing the need to focus on the links between deprivation and the aging population specifically.

**Public health implications—What are the key implications or messages for practitioners, policy makers and/or researchers in public health?**

- Understanding the relationships between air pollution and socioeconomic deprivation and health outcomes are crucial for designing effective interventions and improving public policy.

**Abstract:**

Air pollution and socioeconomic deprivation have been independently associated with multiple negative health outcomes, such as depression and increased pain frequency and duration. This study examined the joint influences of air pollution and individual- and neighborhood-level deprivation on depression and pain. A total of 1113 adults (aged 18–88 years) completed a series of surveys about their mental and physical health and living environment. Participants’ geocoded addresses were used to obtain their long-term average exposure to PM_2.5_ and their neighborhood’s socioeconomic status and resource access via the Area Deprivation Index (ADI). We found significant age interactions with PM_2.5_ and several socioeconomic deprivation indicators. Individual-level and neighborhood-level deprivation on their own significantly predicted levels of depression and pain. We also found that relative deprivation, the measure of an individual’s socioeconomic deprivation compared to their neighborhood, was significantly associated with experiences of both depression and pain. These findings are important to understanding the impacts of environmental stressors, particularly on the aging population, which may contribute to improved interventions and public policy targeting the detrimental associations of environmental stressors with human health.

## 1. Introduction

The negative effects of air pollution exposure beyond respiratory conditions have progressively come to light, particularly when it comes to human health. PM_2.5_ refers to fine particulate matter with a diameter of ≤2.5 µm that consists of solid and liquid materials such as soot and dust [1]. It is particularly harmful because of its ability to be easily inhaled and enter the pulmonary system [2]. Various studies, both in rodents and humans, have connected its unique properties to inflammation in the respiratory system, contributing to respiratory and cardiovascular impairment and disease [2,3,4].

It has become increasingly established that these negative health outcomes can extend to the brain. Prolonged exposure of PM_2.5_ to the respiratory system can induce chronic inflammation and oxidative stress, releasing inflammatory factors into the bloodstream that can cross the blood–brain barrier [5]. PM_2.5_ can also directly access the brain via the olfactory nerve, further breaking down the blood–brain barrier and causing inflammation and damage to the brain tissue [5]. PM_2.5_-induced damage, especially long-term, can lead to cognitive decline in older adults, increased mental illness symptoms, and worsened pain intensity and duration [6,7,8,9]. Due to these alarming risks, learning more about the role air pollution plays in human health is crucial to effectively treating health problems.

While PM_2.5_ levels have been increasing across the United States, PM_2.5_ concentrations overall tend to be higher in more deprived neighborhoods [10,11]. Socioeconomic deprivation can be differentiated into two main levels, individual and neighborhood, both of which have links with worsened well-being independent of air pollution. Individuals who are deprived of resources compared to others tend to experience worse mental and physical health, such as increased prevalence of depression and suicidal behaviors and increased risk of pain interference [12,13,14,15]. Being more deprived than one’s neighborhood or viewing oneself in that way (commonly referred to as relative deprivation) has unique links to poorer health outcomes and behavioral and social struggles [16,17,18]. Neighborhood deprivation, which is typically characterized by their residents having low socioeconomic statuses and lack of resources, has also independently been associated with these negative health aspects [19]. Deprived neighborhoods have been linked to poor cardiovascular health, diabetes, and other diminished health outcomes [16,20]. Worsened experiences of pain and increased depressive symptoms have also been observed in disadvantaged areas [21,22,23,24,25]. Due to these issues and the association of deprivation with more polluted areas, it is critical to examine deprivation factors in combination with air pollution [10].

While air pollution and socioeconomic deprivation are both associated with negative health outcomes, including their combined association with increased cognitive decline in older adults [10,26,27], little research has been done on their joint association with health in general. Our study addresses this gap by looking at the joint influences of air pollution and socioeconomic deprivation on mental and physical health. Specifically, we investigated if differing levels of socioeconomic deprivation are negatively linked with air pollution. For this study, we chose to focus on depression and pain as measures of mental health and physical health, respectively, since they are some of the most studied outcomes alongside air pollution and deprivation, and they also have associations with cognitive and socioeconomic burden [28,29]. Due to decreased cognitive function and increased likelihood of physical conditions causing pain in older adults, we also examined if the correlations with environmental stressors were age-dependent. Additionally, individual-level and neighborhood-level deprivation were examined to account for both individual- and context-level impacts of deprivation. Our analyses focused on three primary questions, which were pre-registered at the outset of the study (Available online: https://osf.io/q8ydb). First, do air pollution and socioeconomic deprivation interact to contribute to an individual’s experiences of depression? Second, do air pollution and socioeconomic deprivation interact to contribute to an individual’s experiences of pain? Third, does being more or less deprived than one’s surrounding environment influence levels of depression and pain?

## 2. Materials and Methods

### 2.1. Study Sample

This study gathered data from 1450 participants (aged 18–88 years) located primarily in the Philadelphia metropolitan area. Informed consent was obtained from all participants involved in the study. Recruitment was conducted through the Temple University Neuroeconomics Laboratory website, social media advertisements, and referrals from past participants. Participants were recruited to resemble the racial and ethnic demographics of the Philadelphia metropolitan area and to be evenly representative of younger and older adults. Participants completed a screener as part of a broader study using functional magnetic resonance imaging (fMRI) to investigate social and financial reward processing across the lifespan [30]. This screener included a series of detailed online questionnaires about participants’ current living arrangements, living conditions, and health experiences via REDCap, and participants were able to complete the surveys at their own pace. Participants were compensated for their time, and their data was scored prior to analysis. This study was approved by the Temple University Institutional Review Board (#28455, 14 October 2021).

The most recent set of complete responses for participants with multiple responses were included to ensure their most current reported address was analyzed. Participant addresses were geocoded using Geocodio (v1.12), an online geocoding service, which provided their coordinates with a corresponding accuracy score (Available online: https://www.geocod.io/, accessed on 21 April 2026). We excluded anyone with incomplete or incorrect address data that resulted in coordinates below 99% accuracy. Participants who provided an address outside of Pennsylvania, New Jersey, Delaware, Maryland, or New York were excluded. They were also excluded if they had missing data for relevant measures outlined below or for failing attention check items. The final sample consisted of 1113 participants, the majority of which identified as female (62.7%) and white (55.8%). Their ages ranged from 18 to 88 years (M = 37.91). Full demographic information for the sample can be found in Table 1.

### 2.2. Measures

#### 2.2.1. Air Pollution Estimations

Air pollution was measured in terms of mean PM_2.5_ concentrations (µg/m^3^) present annually in participants’ neighborhoods, using a spatial resolution of 0.01° × 0.01° (approximately 1.11 km × 1.11 km), with grid cells varying slightly by latitude [31]. Participants’ geocoded addresses were used to retrieve their mean total PM_2.5_ concentrations via the Atmospheric Composition Analysis Group’s North American Regional Estimates with Composition (V5.NA.05/V5.NA.05.02) (Available online: https://sites.wustl.edu/acag/surface-pm2-5/#V5.NA.04.02, accessed on 4 March 2026). These are satellite-derived PM_2.5_ raster datasets that combine aerosol optical depth information via various satellite instruments with simulation and monitor data to create a comprehensive estimation of ground-level PM_2.5_ concentrations [31]. Due to seasonal changes in pollutant concentrations [32,33] and unusual reductions in PM_2.5_ emissions during the COVID-19 pandemic [34,35], we averaged together the annual mean PM_2.5_ concentrations from 2012–2022 to more accurately estimate pollutant trends. We then used point-based extraction (nearest neighbor) to provide an estimate for long-term pollutant exposure at each participant’s address coordinates.

#### 2.2.2. Deprivation Measures

Neighborhood deprivation was measured as a neighborhood ranking from 1 (least disadvantage) to 100 (most disadvantage) using the U.S. Health Resources and Services Administration Area Deprivation Index (ADI). This index takes into consideration income, education, employment, and housing quality via a total of 17 census-based indicators and ranks the socioeconomic status of neighborhoods at the census tract block group level, making it a more extensive measure of neighborhood deprivation than other indices [19]. Based on their given addresses, we matched participants with their national ADI rank via 9-digit ZIP codes.

To measure individual deprivation, we used the U.S. Index of Socioeconomic Deprivation for Individuals (USiDep), which captures participants’ perceived personal socioeconomic deprivation during the past 12 months. This 8-item survey asks about financial assistance received in the past year, unemployment, and cutting back on necessities to save money [36]. Examples of items include “Being forced to buy cheaper food,” “Obtaining help from a community organization,” and “Unemployment” [36]. Participants answered yes/no for each item, and their responses were summed and converted to an ordinal value from 1 (least deprived) to 5 (most deprived) [36].

In addition to these measures, we calculated another measure of an individual’s socioeconomic standing relative to their neighborhood, as prior research suggests that contextual, neighborhood associations may be dependent on individual-level characteristics and prior experiences [37,38]. In this study, relative deprivation was operationalized as the difference between participants’ normalized USiDep scores and normalized national ADI ranks. USiDep scores and ADI ranks were converted to z-scores. ADI was then subtracted from USiDep, resulting in a composite measure of how deprived a participant is compared to their neighborhood. A positive relative deprivation value indicates that an individual is more deprived than their neighborhood, while a negative relative deprivation value indicates an individual is less deprived than their neighborhood.

#### 2.2.3. Depression and Pain Measures

Depression and pain, our two dependent variables, were measured using the Patient-Reported Outcomes Measurement Information System (PROMIS) survey, where we used raw summed scores to assess reported frequency and severity of depression and pain in the past seven days. The PROMIS depression subscale of this survey contains 4 items to evaluate a participant’s experiences of depressive symptoms [39]. Examples of items include “I felt worthless,” “I felt helpless,” and “I felt depressed” [40]. Answer options are provided on a five-point Likert scale.

Likewise, the PROMIS pain interference subscale of this survey contains 4 items evaluating a participant’s difficulties with daily functioning because of pain [41]. Examples of items include, “How much did pain interfere with your day-to-day activities?”, “How much did pain interfere with work around the home?”, and “How much did pain interfere with your ability to participate in social activities?” [41]. Answer options are provided on a five-point Likert scale.

### 2.3. Analyses

Time-averaged estimations of PM_2.5_ concentrations at participant address coordinate point locations were calculated using ArcGIS Pro (version 3.1.0). To analyze the associations between air pollution, neighborhood deprivation, individual deprivation, relative deprivation, depression, and pain (H1.1-H3.2), we performed multiple linear regression analyses using R Studio (version 4.5.1). ChatGPT-5 was used to assist with code generation for analysis and visualization. Statistical significance was determined with an alpha level of 0.05, and the Bonferroni correction was used to control for familywise multiple comparisons. All analyses controlled for age, gender, and race/ethnicity. We also planned to control for depression and pain in each analysis if those two variables were highly correlated. Effect size was evaluated using Cohen’s f^2^ to determine if effects were small (f^2^ = 0.02), medium (f^2^ = 0.15), or large (f^2^ = 0.35) [42].

Exploratory analyses were conducted with our research questions using a subset of our sample that indicated how long they have lived at their provided addresses. For this, exclusions were made for participants that did not provide a response to the question “What date did you move to your current address?” in our screener. Participants who indicated having moved to their given address in 2021 or later were excluded to control for the potential confound of living at a semi-permanent address. The remaining participants reported current residential times ranging from 5 to 62 years (n = 534, M_time_ = 16.49).

Exploratory analyses also assessed whether participants’ perceptions of their subjective social status (represented by the MacArthur Scale of Subjective Social Status) provided a stronger association with air pollution and PROMIS depression and pain scores than USiDep and our relative deprivation calculation. Additionally, we tested if relative deprivation could be a moderator of the relationships between air pollution and the PROMIS outcomes. Due to the wide range of ages in our sample, we also examined if our findings were moderated by age.

For both PROMIS measures, our analyses used raw summed scores over T-scores for transparency and simplicity. We have included our main models using the T-scores in the Supplement to show the minimal impact this had on our results (Supplementary Table S4). Additionally, we planned to adjust for one when investigating the other to isolate outcome-specific variance. This mutual adjustment aligns with our pre-registration, but we recognize that overadjustment is a valid concern. Therefore, results from unadjusted models can be found in the Supplement (Supplementary Table S5), confirming that our conclusions are unchanged and ruling out collider-induced distortion.

### 2.4. Deviations from Pre-Registration

This was a correlational observational study with a between-subjects design, which was pre-registered on OSF (Available online: https://osf.io/q8ydb). Although our main analyses remained consistent with our pre-registration, our exploratory analyses contain deviations. We examined age as a moderator for all of our analyses, both for the main sample and our sample subset, to determine if age was driving the associations between environmental stressors and health outcomes.

## 3. Results

### 3.1. PM_2.5_ Concentration Estimations

**Figure 1:**
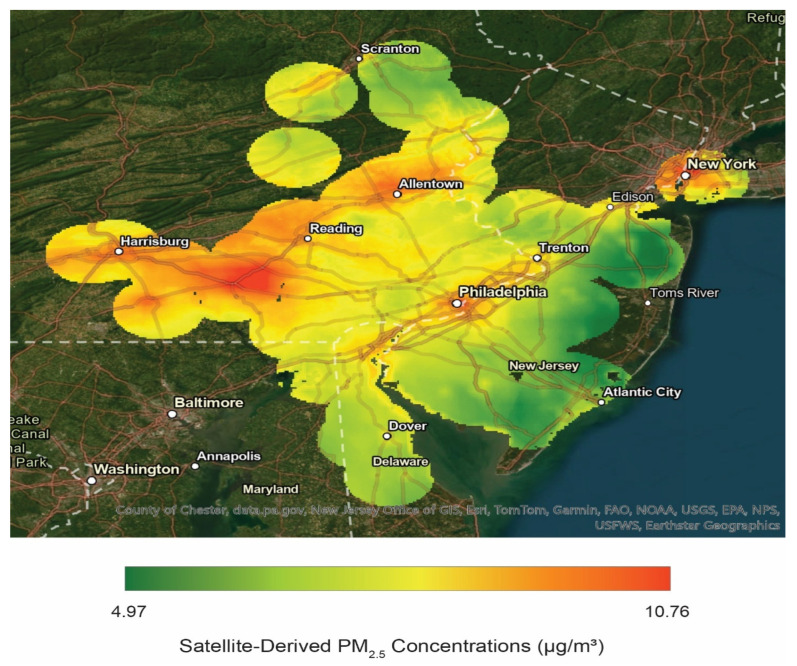
Map of the time-averaged annual PM_2.5_ concentrations from 2012–2022 for the study region covering the sample residential locations. Our map displays high spatial variability in PM_2.5_ concentration throughout the study region, ensuring representation of both high and low concentrations within our sample.

The range of average PM_2.5_ concentrations in our sample was 4.97–10.76 µg/m^3^, which is consistent with previous findings of PM_2.5_ trends in the Philadelphia Metropolitan area [43]. A map of the PM_2.5_ concentrations in our full sample is depicted in Figure 1, and a more detailed map of concentrations in Philadelphia specifically (where most of our participants reported living) can be found in the Supplement (Supplementary Figure S1). Areas with the highest concentrations were around major cities and in regions with high industrial and agricultural activity, which are displayed in red. Areas with the lowest concentrations are displayed in green. We selected 2012–2022 as our timeframe to focus on chronic exposure and reduce sensitivity to short-term fluctuations and anomalous trends (e.g., the COVID-19 pandemic, major wildfires). As a sensitivity analysis, we tested alternative averaging windows (see Supplementary Tables S1–S3) to show that results were materially unchanged.

### 3.2. Predicting PROMIS Depression Scores from PM_2.5_, ADI, and USiDep

Because PROMIS depression and pain scores were correlated (*p* < 0.001 for both regressions), we controlled for one variable when examining the associations with the other. Age, gender, and race/ethnicity were also used as covariates for all models. All analyses controlled for multiple comparisons, and full report of the analyses (including both corrected and uncorrected *p*-values) are reported in Table 2 and Table 3. All models were estimated using the lm function from the stats package in R.

We first investigated whether depressive symptoms were associated with environmental and neighborhood-level disadvantage. We predicted that both PM_2.5_ (air pollution) and ADI scores (neighborhood deprivation) would be positively associated with PROMIS depression scores (Hypothesis 1.1) and that their interaction would predict increased symptom severity (Hypothesis 1.2). ADI scores were revealed to be a significant predictor of PROMIS depression scores (β = 0.016, *t*(1081) = 3.29, *p* = 0.001 (uncorrected), *p* = 0.003 (Bonferroni-corrected), f^2^ = 0.14), implying that residing in a more deprived neighborhood may increase symptoms of depression. In contrast, there was no effect of PM_2.5_ on depression and no interaction effect between PM_2.5_ and ADI on depression. In a separate model, we explored whether these correlations depended on age. In a three-way interaction with PM_2.5_, ADI, and age, regressed onto PROMIS depression scores, we did not find a significant interaction between ADI and age after adjusting for multiple comparisons, indicating that associations of neighborhood deprivation with depressive symptoms do not differ with age.

**Figure 2:**
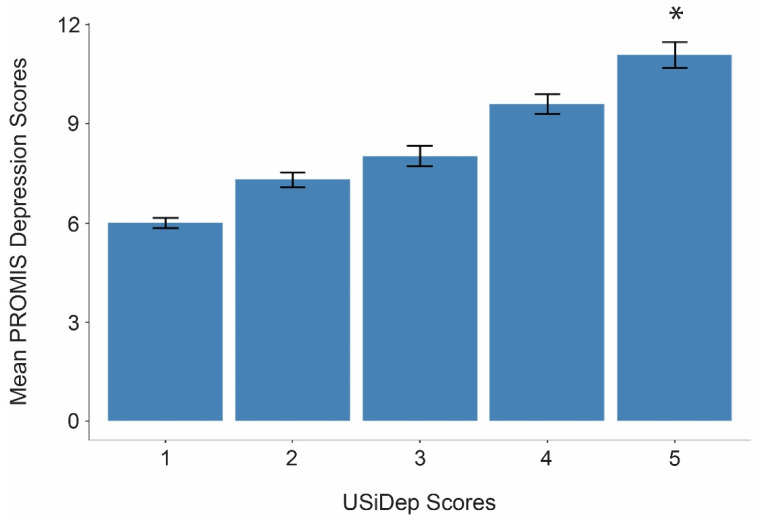
Bar graph with error bars depicting mean PROMIS depression scores for each USiDep score and an asterisk indicating significance. Age, gender, and race/ethnicity were used as covariates. USiDep scores at the highest level (5) were significant predictors of PROMIS depression scores (*p* < 0.001), indicating that experiencing higher levels of individual deprivation are associated with higher levels of depressive symptoms in everyday life.

With evidence that neighborhood-level deprivation is associated with depressive symptoms, we next tested whether individual-level deprivation, measured via USiDep, moderated the association between PM_2.5_ and depressive symptoms (Hypothesis 1.3). Though we did not find a significant interaction between PM_2.5_ and USiDep, our analysis revealed a positive association between USiDep and depression (β = 0.91, *t*(1104) = 10.88, *p* < 0.001 (uncorrected), *p* < 0.001 (Bonferroni-corrected), f^2^ = 0.49) (see Figure 2). We tested this relationship with the subset of our sample that reported the date they moved to their current addresses to see if length of time spent living in an area was a potential confound. Again, we found that increased USiDep scores were positively correlated with PROMIS depression scores (β = 0.90, *t*(505) = 7.66, *p* < 0.001 (uncorrected), *p* < 0.001 (corrected), f^2^ = 0.54), suggesting that regardless of how long an individual lives in their neighborhood, their levels of depression are still highly associated with their individual deprivation status. However, age moderated the relationship between USiDep and depression only after controlling for residential time, with a significant interaction between USiDep and age (β = −0.02, *t*(505) = −2.64, *p* = 0.008 (uncorrected), *p* = 0.02 (Bonferroni-corrected), f^2^ = 0.12). This indicates that residential time may contribute to worsened links between individual deprivation and levels of depression in older adults specifically.

### 3.3. Predicting PROMIS Pain Scores from PM_2.5_, ADI, and USiDep

We then investigated whether pain interference was associated with environmental and socioeconomic disadvantage, predicting that both PM_2.5_ and ADI would be positively associated with PROMIS pain scores (Hypothesis 2.1). Unlike with depression, ADI was not a significant predictor of pain, indicating that while there is evidence for neighborhood deprivation affecting mental health, the same cannot be said for physical health. PM_2.5_ continued to not show a significant effect in our full sample. However, after controlling for residential time, PM_2.5_ significantly predicted PROMIS pain scores (β = 0.59, *t*(505) = 2.68, *p* = 0.009 (uncorrected), *p* = 0.03 (Bonferroni-corrected), f^2^ = 0.17), providing evidence that the correlation between PM_2.5_ and pain interference levels may manifest to a greater degree over the long term.

We also tested if neighborhood deprivation moderated the association between air pollution and pain (Hypothesis 2.2); we found no main effects, and there was no interaction between PM_2.5_ and ADI. A separate model including age found an interaction between ADI and age when the same variables were regressed onto PROMIS pain scores (β = 0.001, *t*(1078) = 2.86, *p* = 0.004 (uncorrected), *p* = 0.01 (Bonferroni-corrected), f^2^ = 0.09), implying that older adults who are particularly vulnerable to increased pain levels are likely to live in a disadvantaged neighborhood.

Although our next model did not support a moderating effect of objective individual deprivation on the association between PM_2.5_ and pain (Hypothesis 2.3) and our main effect of PM_2.5_ did not survive the Bonferroni correction, we did find a significant main effect of USiDep. Higher individual deprivation was associated with greater pain interference in this sample (β = 0.59, *t*(1081) = 7.29, *p* < 0.001 (uncorrected), *p* < 0.001 (Bonferroni-corrected), f^2^ = 0.43). In our sample subset controlling for residential time, we found this same relationship (β = 0.62, *t*(505) = 5.27, *p* < 0.001 (uncorrected), *p* < 0.001 (Bonferroni-corrected), f^2^ = 0.43). These results link social disadvantage to pain outcomes, indicating that individual deprivation may be a key social determinant of pain.

Due to the observed significant interactions with age, we tested a three-way interaction between PM_2.5_, USiDep, and age to observe the interplay of these factors on PROMIS pain. This analysis showed that, similar to our findings with ADI, a significant interaction between USiDep and age (β = 0.018, *t*(1078) = 3.91, *p* < 0.001 (uncorrected), *p* < 0.001 (Bonferroni-corrected), f^2^ = 0.12). Even when controlling for residential time, age continued to moderate the relationship between USiDep and pain (β = 0.03, *t*(502) = 4.41, *p* < 0.001 (uncorrected), *p* < 0.001 (Bonferroni-corrected), f^2^ = 0.20), indicating that older adults may have increased associations between their objective individual-level deprivation and pain levels regardless of residential stability.

### 3.4. Relative Deprivation: A Predictor of Depression and Pain

**Figure 3:**
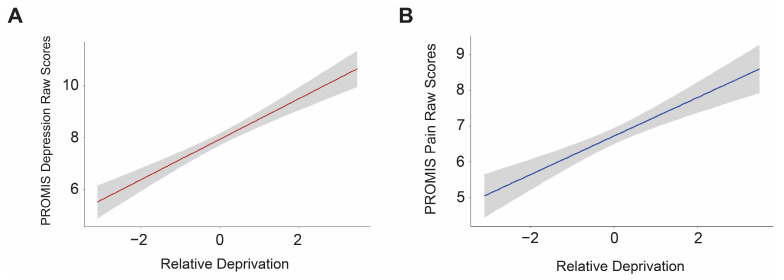
Multiple regression models for (**A**) PROMIS depression vs. relative deprivation and (**B**) PROMIS pain vs. relative deprivation in our main sample (N = 1113). A relative deprivation value less than 0 indicates being less deprived than the neighborhood, and a value more than 0 indicates being more deprived. The shaded areas display the 95% confidence interval. After corrections and controlling for covariates (age, gender, and race/ethnicity), both results were significant (*p* < 0.001), indicating that being more deprived than one’s neighborhood is associated with increased depression and pain symptoms.

Lastly, we tested if relative deprivation predicted depression and pain scores. For these models, ADI and USiDep were not included as covariates to avoid risk of multicollinearity. We hypothesized that relative deprivation would be positively associated with PROMIS depression (Hypothesis 3.1). Our findings revealed that relative deprivation was a significant predictor of PROMIS depression scores (β = 0.50, *t*(1083) = 5.48, *p* < 0.001 (uncorrected), *p* < 0.001 (Bonferroni-corrected), f^2^ = 0.27) (see Figure 3). After controlling for residential time, we continued to see evidence of relative deprivation being positively associated with depressive symptoms (β = 0.64, *t*(507) = 5.19, *p* < 0.001 (uncorrected), *p* < 0.001 (Bonferroni-corrected), f^2^ = 0.34). Next, we predicted that increased relative deprivation would also be positively associated with increased PROMIS pain scores (Hypothesis 3.2). We tested via linear regression of PROMIS pain onto relative deprivation, and our analysis showed that relative deprivation positively linked to PROMIS pain scores (β = 0.34, *t*(1083) = 3.83, *p* < 0.001 (uncorrected), *p* < 0.001 (Bonferroni-corrected), f^2^ = 0.20) (see Figure 3). Relative deprivation continued to positively predict PROMIS pain scores after controlling for residential time (β = 0.31, *t*(507) = 2.53, *p* = 0.01 (uncorrected), *p* = 0.03 (Bonferroni-corrected), f^2^ = 0.23), further suggesting that being more deprived than one’s environment is linked to poorer health in this sample.

In connection with these findings, we explored if subjective perceptions of social status using the MacArthur Scale of Subjective Social Status provided similar or stronger associations with depression and pain. Just like with objective relative deprivation, subjective relative deprivation was found to be a significant predictor of both depression and pain, alongside a significant interaction with PM_2.5_. Full description of this analysis and results is included in the Supplement (Supplementary Figure S2).

## 4. Discussion

In this study investigating the associations of air pollution and socioeconomic deprivation with health outcomes, we predicted that joint associations between air pollution and the various measures of socioeconomic deprivation would produce the worst outcomes for depressive symptoms and pain interference levels. While we did not observe joint associations between these variables, we did find evidence that worsened depression and pain levels are associated with increased levels of air pollution and socioeconomic deprivation independently, as well as significant interactions with variables such as age. Consistent with the current literature [21,22,36], both neighborhood-level deprivation and objective individual deprivation, measured via the Area Deprivation Index (ADI) and United States Index of Socioeconomic Deprivation (USiDep), were significant predictors of depression. Objective individual deprivation positively predicted pain, consistent with previous findings [14,15]. Contrary to others [21], we did not find evidence of neighborhood deprivation predicting pain levels. Despite this, both neighborhood deprivation and self-reported individual deprivation interacted with age in separate models to predict pain scores, showing that older adults may be more at risk for pain symptoms if they live in disadvantaged neighborhoods and are disadvantaged themselves. Objective individual deprivation also interacted with age to predict depression scores in adults with fairly stable residential histories, adding another risk factor for worsened depressive symptoms in older adults dwelling in the same neighborhood for a long period of time.

Although PM_2.5_ was not a significant predictor of depression and had no joint associations with objective deprivation (USiDep) in our sample as predicted, it did have joint associations with subjective deprivation (MacArthur Scale of Subjective Social Status) on predicting depression scores. This is a novel finding that shows the importance of subjective perceptions in heightening vulnerability to environmental stressors and mental illness. Additionally, because of PM_2.5_’s association only with subjective perceptions of deprivation and not objective deprivation in this sample, it implies that subjective deprivation may be the stronger influence in the relationship between PM_2.5_ and depressive symptoms. This could be because although individuals may be objectively deprived, they vary in their resilience and mindsets surrounding their situation. Making social comparisons and considering oneself lower than others, as represented by the MacArthur Scale, could predispose an individual to other thoughts and behaviors common with depression, which is exacerbated by the association with air pollution. Our study also demonstrated that the correlation between air pollution and pain levels were intensified in individuals who lived in their current residence for longer. After controlling for residential time, PM_2.5_ was a significant predictor of pain levels, matching associations in previous research [8,44]. This result shows the negative long-term implications of consistent PM_2.5_ exposure on experiences of pain.

We also demonstrated that relative deprivation (measured as the difference between normalized USiDep scores and normalized ADI ranking) is significantly linked to depression and pain. As our variable of relative deprivation is a composite of USiDep and ADI, which also had positive associations with depression and pain, our findings are logical and also consistent with prior findings [12,45]. This provides valuable evidence that being more deprived than one’s surroundings is associated with higher reports of depressive symptoms and pain interference levels. Not only that, but subjective relative deprivation via the MacArthur Scale is also connected to depression and pain, showing that individuals that either view themselves as more deprived than others or are objectively more deprived will be more prone to poorer health outcomes. Overall, our findings demonstrate correlations between increased environmental stressors and increased experiences of depression and pain, with varying levels of intensity due to age and residential time.

These results highlight important implications of deprivation on human health. Prior literature investigating individual and relative deprivation used subjective measures, such as the Personal Relative Deprivation Scale, that focus on emotion-based items asking about one’s comparison to others [12]. However, there is a lack of measures that calculate relative deprivation objectively. The composite variable we created using two validated measures of deprivation is a novel way of capturing an individual’s relative deprivation. This measure provides additional confirmation that relative deprivation, whether subjective or objective, is a key player in determining the severity of depression and pain symptoms.

Our study’s strengths also lie in our large sample size and our use of more precise measurement tools for our variables. Our satellite-derived datasets measuring PM_2.5_ consider multiple means of measurement, combining satellite data, monitor data, and simulations to create ground-level estimations [31]. Many studies investigating air pollution use ground monitors as the sole measurement of individuals’ average exposure to PM_2.5_ [8]. However, this method may not be representative of individuals’ true exposure levels as monitors are limited, meaning that some individuals may be categorized under the concentrations of the monitor’s site when in reality, they live far from that area and therefore under different conditions [46]. Our use of a long-term exposure average is also beneficial in accounting for seasonal fluctuations and downplaying the impact of outlier years, like the COVID-19 pandemic. It also allowed us to assess cumulative exposure alongside the residential time lengths we had available. For our deprivation measures, we were able to capture a more comprehensive approximation of socioeconomic status through our inclusion of ADI, USiDep, the MacArthur Scale of Subjective Social Status, and relative deprivation, which takes into account objective and subjective measures as well as individual-level and neighborhood-level evaluations.

Despite the strengths of our study, there are still limitations. We did not have access to many of the participants’ full residential histories, only their current addresses, which would have provided a more accurate summary of their long-term PM_2.5_ exposure. Having detailed residential histories would reduce the risk of exposure misclassification, although any exposure misclassification that occurred in this sample likely just attenuated our associations. Additionally, participants may be commonly exposed to pollutants beyond their residential area, such as through public transportation or traveling, which can be just as problematic for health [47,48]. Future studies should build upon this study’s findings and track participants’ PM_2.5_ exposures continuously, whether through wearable PM_2.5_ monitors or other means. These high-precision estimates may reveal a more detailed picture of the impact of PM_2.5_ on depression and pain levels. Participants also have varying access to resources, such as green spaces and healthcare facilities, which may be mitigating factors between deprivation and health [49,50]. These factors should be considered in the future to clarify these relationships. Future studies could also explore different aspects of mental and physical health rather than depression and pain interference. Additionally, we acknowledge that perceiving oneself as more deprived may bias self-reports of depression and pain to be more severe. Therefore, future studies should expand the relative deprivation measure to incorporate more objective indicators, such as directly comparing individual income to the median income of one’s neighborhood, to combat self-report bias. Lastly, we emphasize that this is a cross-sectional study so our results may reflect reverse causation (e.g., individuals with depression may differentially move into more deprived neighborhoods). Future studies should examine our results longitudinally to further investigate age effects and develop experimental conditions to determine causality.

## 5. Conclusions

This study highlights the importance of considering multiple forms of socioeconomic deprivation when assessing mental and physical health. Our findings suggest that individual-level and neighborhood-level deprivation may have distinct associations and that discrepancies between the two warrant closer attention. More broadly, our findings fit with emerging evidence that environmental and community-level conditions may shape a wider range of aging-relevant behavioral outcomes, including more impulsive choice under higher residential PM_2.5_ exposure [51] and greater risk for financial exploitation in contexts of reduced community-level social cohesion [52]. Future research should clarify the relative contribution of each form of deprivation and continue to examine how these social stressors interact with environmental exposures, such as air pollution. As pollution and socioeconomic inequality continue to rise, understanding their combined implications on health will be essential for designing effective public health interventions and policies that reduce the burden of environmental and social disadvantage.

## Figures and Tables

**Table 1:** Demographic Information.

Category	n	%
Gender		
Male	372	33.4%
Female	698	62.7%
Non-binary	42	3.8%
Prefer not to respond	1	0.09%
Race		
American Indian/Alaskan Native	11	1.0%
Asian	96	8.6%
Native Hawaiian/Pacific Islander	3	0.3%
Black/African American	270	24.3%
White	621	55.8%
Two or more races, or other	72	6.5%
Prefer not to respond	40	3.6%
Ethnicity		
Hispanic or Latino	136	12.2%
Not Hispanic or Latino	904	81.2%
Ethnicity other than Hispanic or Latino	46	4.1%
Prefer not to respond	27	2.4%

Summary reflects participants aged 18–88 years old in the final sample (N = 1113). Gender, race, and ethnicity were self-reported.

**Table 2:** Complete summary of all model results in our full sample (N = 1113). Increased socioeconomic deprivation, both on the community and individual levels, was heavily tied to worsened health outcomes.

		Outcome Variable									
		PROMIS Depression					PROMIS Pain				
Model	Predictor	β	t	*p* (Uncorrected)	*p* (Corrected)	Cohen’s f^2^	β	t	*p* (Uncorrected)	*p* (Corrected)	Cohen’s f^2^
Main effects	Depression	-	-	-	-	-	0.39	15.5	<0.001 ***	<0.001 ***	0.46
	Pain	0.46	15.5	<0.001 ***	<0.001 ***	0.44	-	-	-	-	-
	Age	−0.03	−4.02	<0.001 ***	<0.001***	0.13	0.05	6.52	<0.001 ***	<0.001 ***	0.22
	Relative deprivation	0.5	5.48	<0.001 ***	<0.001 ***	0.27	0.34	3.83	<0.001 ***	<0.001 ***	0.2
2-way interactions	PM_2.5_ × ADI	−0.008	−1.2	0.23	0.69	0.04	0.008	1.25	0.21	0.63	0.04
	PM_2.5_	−0.16	−0.93	0.35	>0.99	0.02	0.14	0.92	0.36	>0.99	0.02
	ADI	0.016	3.29	0.001 ***	0.003 ***	0.14	0.008	1.7	0.09	0.27	0.17
	PM_2.5_ × USiDep	0.072	0.74	0.46	>0.99	0.02	−0.14	−1.48	0.14	0.42	0.05
	PM_2.5_	−0.047	−0.348	0.73	>0.99	0.02	0.01	0.08	0.94	>0.99	0.02
	USiDep	0.91	10.88	<0.001 ***	<0.001 ***	0.49	0.59	7.29	<0.001 ***	<0.001 ***	0.43
	PM_2.5_ × Relative deprivation	0.08	0.69	0.49	>0.99	0.02	-	-	-	-	-
	Relative deprivation × Age	0.002	0.39	0.7	>0.99	0.01	0.005	0.91	0.36	>0.99	0.03
3-way interactions	PM_2.5_ × ADI × Age	0.0002	0.55	0.58	>0.99	0.02	−0.0001	−0.33	0.74	>0.99	0.01
	ADI × Age	−0.0006	−2.23	0.02 *	0.06	0.07	0.001	2.86	0.004 **	0.01 **	0.09
	PM_2.5_ × USiDep × Age	<0.001	<0.001	>0.99	>0.99	<0.001	0.002	0.33	0.75	>0.99	0.01
	USiDep × Age	−0.01	−1.47	0.14	0.04	0.12	0.018	3.91	<0.001 ***	<0.001 ***	0.12

Note. β = standardized beta coefficient. Corrections for multiple comparisons were made using Bonferroni. Demographic covariates (age, gender, race/ethnicity, and PROMIS depression or PROMIS pain) were included in the models but are not displayed in this table. * *p* < 0.05, ** *p* < 0.01, *** *p* < 0.001.

**Table 3:** Complete summary for all model results in the subset of our sample controlling for residential time (N = 534). Similar to the full sample, increased socioeconomic deprivation was frequently linked to worsened health outcomes.

		Outcome Variable									
		PROMIS Depression					PROMIS Pain				
Model	Predictor	β	t	*p* (Uncorrected)	*p* (Corrected)	Cohen’s f^2^	β	t	*p* (Uncorrected)	*p* (Corrected)	Cohen’s f^2^
Main effects	Depression	-	-	-	-	-	0.4	10.54	<0.001 ***	<0.001 ***	0.46
	Pain	0.44	10.54	<0.001 ***	<0.001 ***	0.46	-	-	-	-	-
	Age	−0.04	−4.3	<0.001 ***	<0.001 ***	0.23	0.03	2.86	0.004 **	0.012 *	0.16
	Relative deprivation	0.64	5.19	<0.001 ***	<0.001 ***	0.34	0.31	2.53	0.01 **	0.03 *	0.23
2-way interactions	PM_2.5_ × ADI	−0.005	−0.54	0.59	>0.99	0.02	0.02	1.76	0.08	0.24	0.08
	PM_2.5_	−0.008	−0.033	0.97	>0.99	0.06	0.59	2.68	0.009 **	0.03 *	0.17
	ADI	0.004	0.49	0.62	>0.99	0.08	0.006	0.89	0.38	>0.99	0.1
	PM_2.5_ × USiDep	0.11	0.83	0.41	>0.99	0.04	0.009	0.07	0.95	>0.99	<0.001
	PM_2.5_	0.06	0.3	0.76	>0.99	0.07	0.42	2.22	0.03 *	0.09	0.17
	USiDep	0.9	7.66	<0.001 ***	<0.001 ***	0.54	0.62	5.27	<0.001 ***	<0.001 ***	0.43
	Relative deprivation × Age	−0.004	−0.61	0.54	>0.99	0.03	0.01	1.54	0.13	0.39	0.07
3-way interactions	PM_2.5_ × ADI × Age	−0.0005	−0.9	0.37	>0.99	0.04	0.0005	0.99	0.32	0.96	0.04
	ADI × Age	−0.0006	−1.64	0.1	0.3	0.09	0.0007	1.92	0.06	0.18	0.1
	PM_2.5_ × USiDep × Age	0.01	0.89	0.37	>0.99	0.04	0.007	0.86	0.39	>0.99	0.04
	USiDep × Age	−0.02	−2.64	0.008 **	0.02 *	0.12	0.03	4.41	<0.001 ***	<0.001 ***	0.2

Note. β = standardized beta coefficient. Corrections for multiple comparisons were made using Bonferroni. Demographic covariates (age, gender, race/ethnicity, and PROMIS depression or PROMIS pain) were included in the models but are not displayed in this table. * *p* < 0.05, ** *p* < 0.01, *** *p* < 0.001.

## Data Availability

The original contributions presented in this study are included in the article/supplementary materials. Further inquiries can be directed to the corresponding author. The data presented in this study will be made freely available on GitHub after publication.

## Supplementary Materials

The following supporting information can be downloaded at: https://www.mdpi.com/article/10.3390/ijerph23050543/s1, Figure S1: Average PM_2.5_ concentrations from 2012–2022 in Philadelphia and the surrounding area; Figure S2: Johnson-Neyman interaction plot depicting the conditional association between PM_2.5_ and PROMIS depression scores across mean-centered values of the MacArthur Scale of Subjective Social Status; Table S1: Sensitivity analysis of PM_2.5_ concentrations in µg/m^3^; Table S2: Main model results using the average PM_2.5_ concentrations derived from 2012–2014; Table S3: Main model results using the average PM_2.5_ concentrations derived from 2020–2022; Table S4: Main model results using PROMIS T-scores instead of the raw scores; Table S5: Main model results without adjusting for depression when controlling for pain (and vice versa) in our full sample (N = 1113) [53,54].

## Abbreviations

The following abbreviations are used in this manuscript:

PM_2.5_	Particulate matter (diameter of ≤2.5 µm)
ADI	Area Deprivation Index
USiDep	United States Index of Socioeconomic Deprivation for Individuals
