# Supplementary material for: Association of Air Pollution and Socioeconomic Deprivation with Depression and Pain
Source: Int J Environ Res Public Health. 2026 Apr 22;23(5):543. doi: 10.3390/ijerph23050543 (PMC13207363; doi:10.3390/ijerph23050543)
Supplement: Supplementary file 1 [file ijerph-23-00543-s001.zip › ijerph-4180194-supplementary.pdf]

Supplement

Accounting for Medication Access Using PMAS

In our main analyses, we found evidence that individual-level deprivation is linked to increased pain scores. We considered if this relationship could be explained by lack of financial resources, prohibiting access to medication to treat conditions causing pain. We ran analyses with a subset of our sample (N = 593) to evaluate if medication access contributed to the association between individual deprivation and pain using the PROMIS Medication Adherence Scale (PMAS). PMAS includes 9 items regarding medication adherence, with items such as "I took this medication as recommended" and "I did not take this medicine because of the cost," measured on a 5-point Likert scale [53]. For this analysis, we only considered the latter item to specifically measure financial difficulty related to medication usage.

In a model regressing PROMIS pain scores onto PMAS scores, with USiDep, age, gender, and race/ethnicity as covariates, there was no significant effect of medication access on pain, $p = 0.44$, suggesting that the relationship we found between USiDep and PROMIS pain scores cannot be explained by financial access to medication within this sample.

The MacArthur Scale as a Measure of Relative Deprivation

In previous literature, the MacArthur Scale of Subjective Social Status is a common measurement of subjective relative deprivation. This survey presents participants with an image of a ladder with rungs labeled 1-10 starting from the bottom, asking them to rate themselves on the ladder if 1 represents those with the least money, education, and job respectability, and 10 represents those with the most money, education, and job respectability [54].

Because relative deprivation is a measure of how objectively deprived an individual is compared to their neighborhood, we assessed if an individual's subjective perceptions of their social status would be more influential in determining the relationship between air pollution and pain. Using the MacArthur Scale of Subjective Social Status, we tested a model with $PM_{2.5}$, MacArthur Scale scores, and their interaction on PROMIS depression scores. Subjective social status on its own was a significant predictor of PROMIS depression scores, $\beta = -.47$, $t(1081) = -7.71$, $p < 0.001$ ($f^2 = 0.40$), similar to objective relative deprivation. Notably, our results showed a significant interaction between MacArthur Scale scores and $PM_{2.5}$, $\beta = 0.16$, $t(1081) = 2.14$, $p = 0.03$ ($f^2 = 0.07$), indicating that subjective views of deprivation may be more influential than objective deprivation on $PM_{2.5}$-induced sensitivity to depression (Fig. 4).

Additionally, we looked at the interaction between MacArthur Scale scores and $PM_{2.5}$ on PROMIS pain scores. The MacArthur Scale remained a significant predictor when regressed onto pain scores, $\beta = -.37$, $t(1081) = -6.32$, $p < 0.001$ ($f^2 = 0.32$). Although we

found a significant interaction between the MacArthur Scale and $PM_{2.5}$ when regressed onto PROMIS depression, we did not find this result between these predictors and pain, $p = 0.61$ ($f^2 = 0.02$). However, in a three-way model with $PM_{2.5}$, the MacArthur Scale, and age, the MacArthur Scale did significantly interact with age, $\beta = -.014$, $t(1101) = -4.37$, $p < 0.001$ ($f^2 = 0.13$).

$PM_{2.5}$ Sensitivity Analysis Results

For our sensitivity analysis, we tested alternate averaging windows to clarify the validity of our long-term average from 2012-2022. We averaged both the first three years of our data and the last three years, which is depicted in Table S1. Our findings demonstrate $PM_{2.5}$ concentrations in our sample remain stable over time, as our results are consistent between 3-year averages and our 11-year average. Again, while exposure misclassification is a possibility, especially without detailed residential histories from the full sample, any misclassification merely attenuates our results. The subsequent results from our models are presented in Tables S2 and S3 for the 2012-2014 average and the 2020-2022 average. Our findings remain consistent with our original models that used the 11-year average, demonstrating that fluctuations in air pollution over time only marginally change our results.

Using PROMIS T-Scores for Analyses

For the sake of transparency and simplicity, we opted to use the raw summed scores for the PROMIS surveys in our analyses. However, as PROMIS scores are often represented as T-scores, we present the results of our analyses using T-scores in Table S4. Even with a different scoring method on our data, our conclusions remain unchanged.

Running Models Without Mutual Adjustment

In this study, we controlled for depression when predicting pain (and vice versa) because of their high correlation, allowing us to focus on outcome-specific associations. To address the concern of overadjustment, we present our main models without mutual adjustment in Table S5. Our results remained consistent with our adjusted models except for ADI and PROMIS pain. In our adjusted model, ADI was not associated with PROMIS pain, but our unadjusted model here shows ADI as a significant predictor of pain. However, mutual adjustment overall had minimal impact on our results.

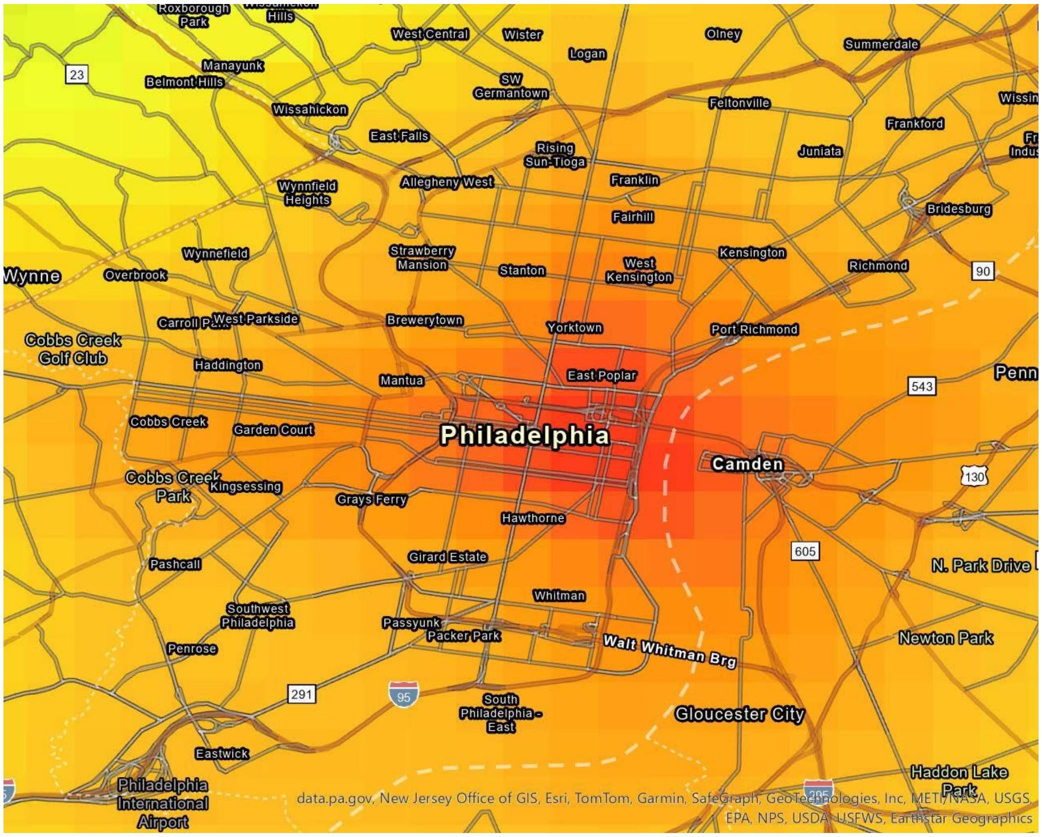

Figure S1. Average $PM_{2.5}$ concentrations from 2012-2022 in Philadelphia and the surrounding area.

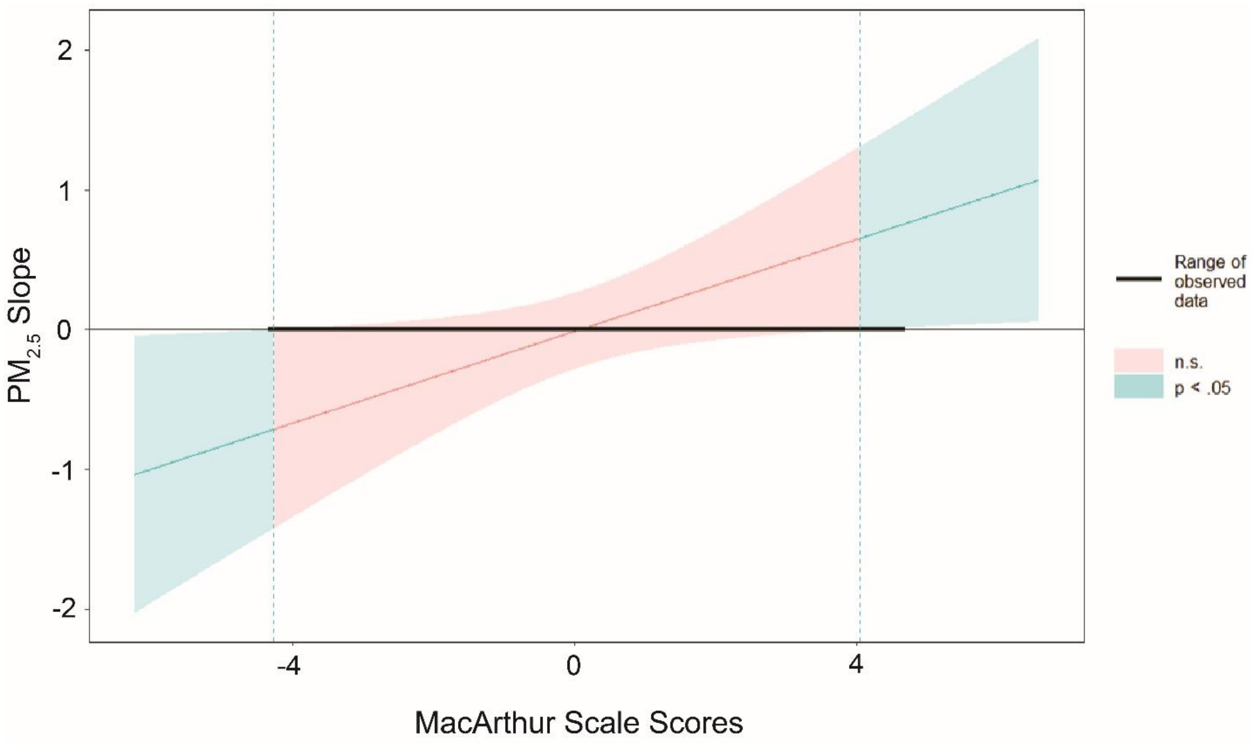

Figure S2. Johnson-Neyman interaction plot depicting the conditional association between $PM_{2.5}$ and PROMIS depression scores across mean-centered values of the MacArthur Scale of Subjective Social Status. The y-axis shows the estimated simple slope for $PM_{2.5}$ predicting depression at each value of the moderator. Values above 0 indicate a positive association between $PM_{2.5}$ and depression, and values below 0 indicate a negative association. The shaded bands represent 95% confidence intervals. Blue regions indicate values of the moderator for which the $PM_{2.5}$ slope is statistically significant at $p < 0.05$, whereas pink regions indicate non-significant values. The black horizontal segment marks the observed range of the moderator in the sample.

Table S1. Sensitivity analysis of $PM_{2.5}$ concentrations in μg/m^3.

2012-2022	4.97 - 10.76
2012-2014	5.43 - 12.13
2020-2022	4.77 - 10.47

Table S2. Main model results using the average $PM_{2.5}$ concentrations derived from 2012-2014.

		Outcome variable									
		PROMIS Depression					PROMIS Pain				
**Model**	**Predictor**	**β**	**t**	***p* (uncorrected)**	***p* (corrected)**	**Cohen's f^2**	**β**	**t**	***p* (uncorrected)**	***p* (corrected)**	**Cohen's f^2**
**Hypothesis 1**	$PM_{2.5}$ × ADI	-0.0052	-0.61	0.54	> 0.99	0.02	0.085	1.09	0.28	0.84	0.03
	$PM_{2.5}$	-0.1	-0.51	0.61	> 0.99	0.02	0.15	0.78	0.43	> 0.99	0.01
	ADI	0.024	4.41	< 0.001***	< 0.001***	0.13	0.017	3.39	< 0.001***	0.002**	0.15
**Hypothesis 2**	$PM_{2.5}$ × USiDep	0.036	0.3	0.76	> 0.99	< 0.01	-0.16	-1.42	0.16	0.48	0.04
	$PM_{2.5}$	-0.039	-0.24	0.81	> 0.99	0.02	0.033	0.22	0.83	> 0.99	0.01
	USiDep	1.24	15.1	< 0.001***	< 0.001***	0.47	0.98	12.54	< 0.001***	< 0.001***	0.4

Note. β = standardized beta coefficient. Corrections for multiple comparisons were made using Bonferroni. Demographic covariates (age, gender, race/ethnicity, and PROMIS depression or PROMIS pain) were included in the models but are not displayed in this table. * $p < 0.05$, ** $p < 0.01$, *** $p < 0.001$.

Table S3. Main model results using the average PM2.5 concentrations derived from 2020-2022.

		Outcome variable									
		PROMIS Depression					PROMIS Pain				
**Model**	**Predictor**	**β**	**t**	***p* (uncorrected)**	***p* (corrected)**	**Cohen's f^2**	**β**	**t**	***p* (uncorrected)**	***p* (corrected)**	**Cohen's f^2**
**Hypothesis 1**	$PM_{2.5}$ × ADI	-0.0062	-0.85	0.4	> 0.99	0.03	0.0042	0.61	0.54	> 0.99	0.02
	$PM_{2.5}$	-0.12	-0.65	0.51	> 0.99	0.02	0.071	0.43	0.67	> 0.99	0.02
	ADI	0.024	4.44	< 0.001***	< 0.001***	0.13	0.017	3.44	< 0.001***	0.002**	0.15
**Hypothesis 2**	$PM_{2.5}$ × USiDep	0.022	0.22	0.83	> 0.99	< 0.01	-0.11	-1.2	0.23	0.69	0.04
	$PM_{2.5}$	-0.052	-0.37	0.71	> 0.99	0.02	-0.02	-0.15	0.88	> 0.99	0.02
	USiDep	1.24	15.11	< 0.001***	< 0.001***	0.47	0.98	12.52	< 0.001***	< 0.001***	0.4

Note: β = standardized beta coefficient. Corrections for multiple comparisons were made using Bonferroni. Demographic covariates (age, gender, race/ethnicity, and PROMIS depression or PROMIS pain) were included in the models but are not displayed in this table. * $p < 0.05$, ** $p < 0.01$, *** $p < 0.001$.

Table S4. Main model results using PROMIS T-scores instead of the raw scores.

		Outcome variable									
		PROMIS Depression					PROMIS Pain				
**Model**	**Predictor**	**β**	**t**	***p* (uncorrected)**	***p* (corrected)**	**Cohen's f^2**	**β**	**t**	***p* (uncorrected)**	***p* (corrected)**	**Cohen's f^2**

**Hypothesis 1**	PM2.5 × ADI	-0.02	-1.2	0.23	0.69	0.04	0.02	1.25	0.21	0.63	0.04
	PM2.5	-0.38	-0.93	0.35	> 0.99	0.02	0.37	0.92	0.36	> 0.99	0.02
	ADI	0.039	3.29	0.001***	0.001***	0.14	0.02	1.7	0.09	0.27	0.17
**Hypothesis 2**	PM2.5 × USiDep	0.17	0.74	0.46	> 0.99	0.02	-0.35	-1.48	0.14	0.42	0.05
	PM2.5	-0.11	-0.35	0.73	> 0.99	0.02	0.026	0.079	0.94	> 0.99	0.02
	USiDep	2.18	10.88	< 0.001***	< 0.001***	0.49	1.51	7.29	< 0.001***	< 0.001***	0.43
**Hypothesis 3**	Relative deprivation	1.21	5.48	< 0.001***	< 0.001***	0.27	0.84	3.83	< 0.001***	< 0.001***	0.2

Note. β = standardized beta coefficient. Corrections for multiple comparisons were made using Bonferroni. Demographic covariates (age, gender, race/ethnicity, and PROMIS depression or PROMIS pain) were included in the models but are not displayed in this table. * p < 0.05, ** p < 0.01, *** p < 0.001.

Table S5. Main model results without adjusting for depression when controlling for pain (and vice versa) in our full sample (N = 1113).

		Outcome variable									
		PROMIS Depression					PROMIS Pain				
**Model**	**Predictor**	**β**	**t**	***p* (uncorrected)**	***p* (corrected)**	**Cohen's f^2**	**β**	**t**	***p* (uncorrected)**	***p* (corrected)**	**Cohen's f^2**
**Hypothesis 1**	PM2.5 × ADI	-0.006	-0.75	0.45	> 0.99	0.02	0.0057	0.82	0.41	> 0.99	0.02
	PM2.5	-0.11	-0.6	0.55	> 0.99	0.02	0.1	0.59	0.56	> 0.99	0.01
	ADI	0.024	4.43	0.001***	0.003***	0.13	0.017	3.41	< 0.001***	< 0.001***	0.15
**Hypothesis 2**	PM2.5 × USiDep	0.028	0.27	0.79	> 0.99	< 0.001	-0.13	-1.31	0.19	0.57	0.04
	PM2.5	-0.049	-0.34	0.73	> 0.99	0.02	-0.005	-0.037	0.97	> 0.99	0.02
	USiDep	-0.028	-3.76	< 0.001***	< 0.001***	0.47	0.98	12.53	< 0.001***	< 0.001***	0.4
**Hypothesis 3**	Relative deprivation	0.77	7.81	< 0.001***	< 0.001***	0.25	0.61	6.72	< 0.001***	< 0.001***	0.19

Note. β = standardized beta coefficient. Corrections for multiple comparisons were made using Bonferroni. Demographic covariates (age, gender, and race/ethnicity) were included in the models but are not displayed in this table. * p < 0.05, ** p < 0.01, *** p < 0.001.
